# Modification of Neurogenic Colonic Motor Behaviours by Chemogenetic Ablation of Calretinin Neurons

**DOI:** 10.3389/fncel.2022.799717

**Published:** 2022-03-03

**Authors:** Jing Feng, Tim J. Hibberd, Jialie Luo, Pu Yang, Zili Xie, Lee Travis, Nick J. Spencer, Hongzhen Hu

**Affiliations:** ^1^Center for the Study of Itch and Sensory Disorders, Department of Anesthesiology, Washington University School of Medicine, St. Louis, MO, United States; ^2^Center for Neurological and Psychiatric Research and Drug Discovery, Shanghai Institute of Materia Medica, Chinese Academy of Sciences, Shanghai, China; ^3^College of Medicine and Public Health, Centre for Neuroscience, Flinders University, Adelaide, SA, Australia

**Keywords:** colonic motor complex, IPAN, enteric nervous system, large intestine, colon, sensory neuron, peristalsis

## Abstract

How the enteric nervous system determines the pacing and propagation direction of neurogenic contractions along the colon remains largely unknown. We used a chemogenetic strategy to ablate enteric neurons expressing calretinin (CAL). Mice expressing human diphtheria toxin receptor (DTR) in CAL neurons were generated by crossing *CAL-ires-Cre* mice with *Cre*-dependent *ROSA26-DTR* mice. Immunohistochemical analysis revealed treatment with diphtheria toxin incurred a 42% reduction in counts of Hu-expressing colonic myenteric neurons (*P* = 0.036), and 57% loss of CAL neurons (comprising ∼25% of all Hu neurons; *P* = 0.004) compared to control. As proportions of Hu-expressing neurons, CAL neurons that contained nitric oxide synthase (NOS) were relatively spared (control: 15 ± 2%, CAL-DTR: 13 ± 1%; *P* = 0.145), while calretinin neurons lacking NOS were significantly reduced (control: 26 ± 2%, CAL-DTR: 18 ± 5%; *P* = 0.010). Colonic length and pellet sizes were significantly reduced without overt inflammation or changes in ganglionic density. Interestingly, colonic motor complexes (CMCs) persisted with increased frequency (mid-colon interval 111 ± 19 vs. 189 ± 24 s, CAL-DTR vs. control, respectively, *P* < 0.001), decreased contraction size (mid-colon AUC 26 ± 24 vs. 59 ± 13 gram/seconds, CAL-DTR vs. control, respectively, *P* < 0.001), and lacked preferential anterograde migration (*P* < 0.001). The functional effects of modest calretinin neuron ablation, particularly increased neurogenic motor activity frequencies, differ from models that incur general enteric neuron loss, and suggest calretinin neurons may contribute to pacing, force, and polarity of CMCs in the large bowel.

## Highlights

– Neural mechanisms that determine pacing and propagation direction of neurogenic contractions along the colon remain largely unknown.
– We used chemogenetic techniques to selectively ablate calretinin-expressing neurons in the ENS to determine the functional role of CAL neurons in motility and transit.
– Mice were generated expressing human diphtheria toxin receptor (DTR) in CAL neurons by crossing *CAL-ires-Cre* mice with *Cre*-dependent *ROSA26-DTR* mice.
– A reduction of the CAL neuron population by ∼25% increased CMC frequency, decreased contraction amplitude and lacked preferential anterograde migration compared to controls.

## Introduction

In vertebrate large intestine, a number of neurogenic motor patterns occur that require or involve the enteric nervous system (Costa et al., 2019; Spencer and Hu, 2020). In mouse, the colonic motor complex (CMC) is analogous to the cyclic motor complexes in guinea pig colon. This behaviour comprises regularly occurring neurogenic contractions of the longitudinal and circular muscle of the large intestine that propel content over considerable distances and have been recorded from a variety of species, including humans (Bampton et al., 2000; Hagger et al., 2002; Spencer et al., 2012; Dinning P. et al., 2014), mice (Fida et al., 1997; Balasuriya et al., 2016; Hibberd et al., 2017; Spencer et al., 2018), guinea-pigs (Costa and Furness, 1976; Costa et al., 2013), and rabbits (Fida et al., 1997; Powell et al., 2002; Dinning et al., 2012; Costa et al., 2013). It is well-established that CMCs require enteric neurons for coordination as they are immediately abolished by neuronal blockade with tetrodotoxin (TTX) (Wood et al., 1986; Fida et al., 1997; Brierley et al., 2001; Powell et al., 2002; Spencer and Bywater, 2002; Powell and Bywater, 2003) or by blockade of cholinergic nicotinic receptors (Bush et al., 2000; Brierley et al., 2001; Powell et al., 2002; Powell and Bywater, 2003; Powell et al., 2003), the primary mode of fast neurotransmission among enteric neurons (Galligan, 2002). All enteric neurons, including those that generate CMCs, can be classified by their neurochemical content. Much is known about the different neurochemical classes of enteric neurons that exist and their relationships to functional classifications (Costa et al., 1996; Sang and Young, 1996, 1998; Lomax and Furness, 2000). However, complex motor behaviours and neurochemical classification are typically studied in isolation. Thus, little is known about how neurochemical classes of enteric neurons contribute to complex neurogenic motor behaviours.

A major population of neurons in the large intestine of mice express the neurochemical marker, calretinin which comprises about 30–50% of myenteric neurons (Sang and Young, 1996; Musser et al., 2015). Calretinin neurons comprise myenteric motor neurons, interneurons and importantly, the vast majority of putative primary afferent neurons that also express calcitonin gene related peptide (CGRP) (99%) (Sang and Young, 1996; Sang and Young, 1997; Sang and Young, 1998; Furness et al., 2004). These are presumptively analogous to the Dogiel type II cells that have been referred to as intrinsic primary afferent neurons (IPANs) and have been shown to be directly mechanosensitive in guinea pig (Kirchgessner et al., 1992; Kunze et al., 1998; Kunze et al., 2000) and in the mouse ileum (Mao et al., 2006). Although, there is also evidence of direct mechanosensitivity among populations of myenteric neurons with Dogiel Type I morphology (i.e., interneurons or motor neurons; Spencer and Smith, 2004; Mazzuoli and Schemann, 2009; Mazzuoli and Schemann, 2012; Mazzuoli-Weber and Schemann, 2015), it has been suggested that IPANs, most of which express calretinin, are the first neurons to activate myenteric neural circuits (Kunze and Furness, 1999).

In this study, we selectively ablated calretinin-expressing neurons in adult mice by expressing the human diphtheria toxin receptor (DTR) in calretinin neurons. This allowed us for the first time to characterise the effects in the large bowel of ablation of calretinin-expressing neurons on motility and *in vitro* transit in adult mice, without concerns of genetic compensation. The results suggest that enteric calretinin neurons may modulate pacemaker frequency and propagation direction of CMCs.

## Materials and Methods

### Animals

**FIGURE 1:**
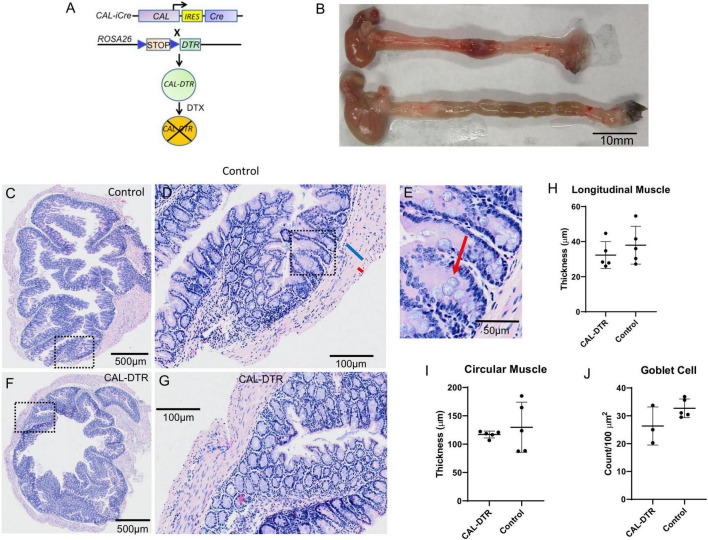
Morphology of control and CAL-DTR colons following H&E staining. **(A)** Gene construct diagram for generation of mice expressing human diphtheria toxin receptor in cells that express the calbindin-2 gene product, calretinin. **(B)** Representative examples of whole CAL-DTR and control colons, showing the reduced length in CAL-DTR mice. **(C)** H&E section of colon from control mouse. Panel **(D)** shows expanded region of the mucosa from the dotted square region in panel **(C)**. The blue bar indicates the thick circular muscle and the smaller red bar the much thinner longitudinal muscle (identified by different shaped nuclei). Panel **(E)** shows an expanded region from panel **(D)** with red arrow indicating a goblet cell. **(F)** H&E section from a colon obtained from a CAL-DTR mouse. Panel **(G)** shows an expanded region of mucosa from the dotted square region in panel **(F)**. Note the similarity (i.e., lack of overt inflammation) in mucosa in panel **(G)** as in control mouse in panel **(D)**. Panel **(G)** shows circular muscle thicknesses in control and CAL-DTR mice. Panel **(H)** shows longitudinal muscle thicknesses in control and CAL-DTR mice. Panel **(I)** shows circular muscle thickness in control and CAL-DTR mice. Panel **(J)** shows goblet cell densities in control and CAL-DTR mice. Each dot point represents a mean value from each animal studied. A total of *N* = 5 control and *N* = 3 DTX mice were used in analysis. There was no significant difference between any of the parameters measured between control and CAL-DTR mice.

Calb2-IRES-Cre [B6(Cg)-*Calb2^TM 1(cre)Zjh^*/J] [stock #010774 (Taniguchi et al., 2011)] and Rosa-DTR mice [stock #016603 (Buch et al., 2005)] obtained from Jackson Laboratories (Bar Harbor, ME, United States) were crossbred (Figure 1A). Resulting *CAL*^cre+^*-DTR*^f/f^** progeny co-expressed human DTR with the Calb2 gene product, calretinin. All mice were housed under a 12-h light/dark cycle with food and water provided *ad libitum*. Age-matched, *Cre*^–^ littermates were used as control in all experiments. Mice ranged 8–12-weeks old in all experiments. Animal studies are reported in compliance with the ARRIVE guidelines, as well as the guidelines of the National Institutes of Health and the International Association for the Study of Pain and were approved by the Animal Studies Committee at Washington University School of Medicine and conform to the principles and regulations as described in the Editorial by Grundy (2015).

### Diptheria Toxin Treatment

Depletion of calretinin neurons was accomplished by using intraperitoneal injections of diphtheria toxin (diptheria toxin (DTX); 600 ng per mouse) every 48 h for a total of 3 treatments to both *cre*^–^ control and *cre*^+^ *CAL-DTR* mice (Luo et al., 2018). At least 1 week was allowed between the final DTX treatment and experimentation. Ablation efficiency was confirmed by counting CAL-IR myenteric nerve cell bodies after all experiments.

### Tissue Preparation

The abdomen was immediately opened after the mouse was euthanised and the entire colon from caecum to terminal rectum was removed. In all experiments except the imaging of natural pellet expulsion, the full length of colon was placed in a petri dish filled with carbogen-gassed (95% O_2_/5% CO_2_) Krebs solution (25–30°C; in 10^–3^ M concentrations: NaCl 118; KCl 4.7, NaH_2_PO_4_ 1; NaHCO_3_ 25; MgCl_2_ 1.2; D-Glucose 11; CaCl_2_ 2.5). Whole colon length was measured before emptying by gentle flushing with Krebs solution and removal of the mesentery. For recording natural pellet expulsion *in vitro*, the terminal rectum was occluded by tying off with fine suture thread before the colon was removed from the animal.

### Haematoxylin and Eosin

Colons were fixed in 4% paraformaldehyde in phosphate-buffered saline (PBS), dehydrated in 30% sucrose, and embedded in optimal cutting temperature (OCT) compound. Twelve-micron sections were made using a Leica CM1950 cryostat (Leica Biosystems, Buffalo Grove, IL, United States). Haematoxylin and Eosin (H&E) staining was performed according to standard protocols.

### Immunohistochemistry

Full-length colons were cleared of content and cut along the mesenteric border. Flat sheet preparations were stretched maximally and fixed for 48 h in paraformaldehyde (4% in 0.1 M phosphate buffer, pH 7.0). The mucosa was removed by sharp dissection and the submucosal layer was peeled off the underlying circular muscle intact. Longitudinal muscle—myenteric plexus preparations were obtained by removing the remaining circular muscle. Preparations were cleared using 0.5% Triton-X100 in 0.1 M phosphate buffered saline (PBS; 0.15 M NaCl, pH 7.2; 3 × 10 min washes and then washed in PBS (3 × 10 min washes) followed by incubation.

Whole length preparations of colonic longitudinal muscle—myenteric plexus were incubated for 30 min to 1 h in phosphate buffered saline (PBS) + 0.1% Triton X-100 containing 10% donkey serum, followed by 24 h incubation in a combination of primary antisera at room temperature. Primary antisera were nitric oxide synthase (NOS) (sheep; 1:2000; Emson; Cat. No. K205; RRID: AB_90743), calretinin (goat; 1:2000; Swant; Cat. No. CG1; RRID: AB_10000342), and Hu C/D (mouse; 1:250; Molecular Probes; Cat. No. A21271; RRID: AB_221448). Preparations were then washed in PBS (3 × 10 min), placed in the appropriate combination of secondary antibodies (1:500 donkey anti-sheep FITC, cat no. 713-095-147, RRID: AB_2340719; 1:500 donkey anti mouse CY3, cat. no. 715-165-150, RRID: AB_2340813; 1:100 donkey anti-goat CY5, cat no. 705-175-147, RRID: AB_2340415; Jackson ImmunoResearch Laboratories, Incorporated, PA, United States) for 1 h.

Preparations were then washed with PBS (3 × 10 min) and equilibrated in a series of carbonate-buffered glycerol solutions (50, 70, and 100% solutions; 3 × 10 min) before mounting on glass slides in buffered glycerol (pH 8.6). Preparations were viewed and imaged using an epifluorescence microscope (Olympus IX71, Japan) equipped with discriminating filters to match the fluorophores used (Chroma Technology Co., Battledore, VT). Images were captured by a Roper Scientific camera and AnalySIS Imager 5.0 software (Olympus-SIS, Munster, Germany) *via* 20 × or 40 × water immersion lenses. Images were stored as TIFF files (1392 * 1080 pixels) and optimised for contrast and brightness using Adobe Photoshop (2021 Adobe Systems Software Ireland Ltd., Mountain View, CA, United States) before analysis. CAL-IR and NOS-IR myenteric nerve cell bodies were quantified by performing counts from a minimum 15 randomly selected fields of view taken as approximately equal intervals along the length of the colon. The field of view (FOV) encompassed a region of 465 × 465 μm. Analyses were performed by identifying all Hu-immunoreactive myenteric nerve cell bodies before assessing their NOS and calretinin immunoreactive content. Assessment was performed blinded to preparation genotype. Analysis was performed on micrographs taken using 20 × immersion lens, comprising a 0.216 mm^2^ field of view. Cell counts in CAL-DTR colons were corrected by a factor of 0.757 to account for mean change in colon size. Both corrected and uncorrected counts are presented.

The specificity of CAL-ires-Cre eYFP expression in colon was characterised previously, revealing detectable eYFP in 97 ± 7% of calretinin-immunoreactive nerve cell bodies and conversely, calretinin-immunoreactivity in 98% of eYFP-containing nerve cell bodies (Hibberd et al., 2018a).

### Mechanical and Electrophysiological Recordings

Combined mechanical and electrophysiological recordings were made from full-length colon preparations in an organ bath, similar to that described previously (Hibberd et al., 2018a). The organ bath (volume ∼50 ml) was continuously superfused at ∼5 ml.min^–1^ (36°C). A stainless steel tube (diameter 1 mm) placed through the lumen was fitted at each end into L-shaped barbed plastic connectors that were fixed to organ bath base. The oral and anal ends of colon were tied over the barbed connectors with a fine suture thread. Stainless steel hooks (250 μm diameter) were threaded through the wall of the proximal, middle, and distal colon. A fine suture thread connected each hook to an isometric force transducer (MLT050/D; ADInstruments, Bella Vista, NSW, Australia) and set to a basal tension of 0.5 g to record mechanical activity. Activity was recorded at 1 kHz (PowerLab 16/35, LabChart 8, ADInstruments, Bella Vista, NSW, Australia).

During mechanical recordings, conventional extracellular recordings were made from the serosal surface of the colon. Two Krebs-filled suction electrodes (AgCl, 250 μm) with heat-polished glass capillary tips were used (0.86 mm internal diameter, 1.5 mm outer diameter; cat# 30-0053, Harvard Apparatus, Holliston, MA, United States). Flexible Silastic tubing (Dow Corning Corporation, Midland, MI, United States) connected the glass tip to the AgCl electrode. This enabled electrodes to remain in position during gut movement. Signals were amplified by a factor of 1000 (ISO80; WPI, Sarasota, FL, United States) and recorded at 1 kHz (PowerLab 16/35, LabChart 8, ADInstruments, Bella Vista, NSW, Australia). The signals recorded were in principle assumed to represent extracellular recording of changes in polarity along the surface of the smooth muscle cells. Electrodes are referred as “oral” and “aboral” electrodes in Figures 7D–F. However, the positioning of electrodes was similar to that described previously (Hibberd et al., 2018a), whereby both electrodes were located approximately 10 mm apart, between the mid and distal hooks used for mechanical recordings.

### Natural Pellet Expulsion and Spatio-Temporal Mapping

The full length of colon containing natural pellets was placed into a Krebs-filled, Sylgard-lined organ bath (Sylgard 184, Dow Corning Corporation, Midland, MI, United States). Preparations were held in place without obstructing the passage of pellets using etymological pins [500 μm diameter (Barnes et al., 2014)]. The suture was then cut free and expulsion of natural pellets was observed over the following 30 min. Gut movements were videographed by a camera placed above the organ bath (Carl Zeiss Tessar C920, Logitech International S.A., Lausanne, Switzerland). Videographs were transformed into maps of circumferential gut diameter (DMaps) using the spatio-temporal mapping technique described by Hennig et al. (1999).

### Statistics and Analysis

Spatio-temporal maps were generated from video recordings using computer script for Matlab software (MathWorks Incorporated, Natick, MA, United States). Script was written in-house at Flinders University (Lukasz Wiklendt, Flinders University). DMaps are generated by measuring gut diameter at each point along the preparation in every video frame and converting diameters into grayscale pixels to create a spatiotemporal map of diameter changes (Figure 8). Regions of minimal diameter (contraction) are represented as white pixels, whereas maximal diameter (distension) are represented by black pixels. This method was adapted from what was described in detail elsewhere (Hennig et al., 1999). Diameter maps were analysed manually using PlotHRM software (Lukasz Wiklendt, Flinders University), written in Matlab (Mathworks) and JavaTM (Sun Microsystems, Santa Clara, CA, United States).

Statistical differences were considered significant if *P* < 0.05. Results are expressed as mean ± standard deviation except where otherwise stated. Lower case “*n*” always indicates the number of animals used in a set of experiments. Statistical analysis was performed by ANOVA (one-way or two-way), Student’s two-tailed *t*-test for paired or unpaired data using Prism 6 (GraphPad Software, Inc., La Jolla, CA, United States). All authors had access to the study data and had reviewed and approved the final manuscript.

## Results

### Colonic Length, Muscle Thickness, and Inflammation

Whole colons of DTX-treated CAL-DTR and age-matched control mice were removed and measured. Compared to control, colonic length of DTX-treated CAL-DTR mice was significantly reduced (length 6.75 ± 0.51 cm vs. 5.10 ± 0.50 cm, *n* = 6 and 4, control and CAL-DTR, respectively, *P* = 0.001, independent samples *t*-test). To determine whether the colonic mucosa underwent anatomical and architectural changes after DTX treatment, we compared H&E sections between *Cre*^–^ (control) and *Cre*^+^ (DTX-treated CAL-DTR) mice. Following DTX treatment, the cell structure and mucosal architecture was normal in both cohorts of animals, with no evidence mucosal ulcerations or necrosis (Figure 1). To determine if DTX-treated CAL-DTR mice had inflammation induced by DTX treatment, we compared the number of goblet cells in the colon, between control and DTX-treated CAL-DTR mice. There was no difference in goblet cell density between control (30 ± 2 per 100 μm^2^; *N* = 3) and DTX-treated CAL-DTR colons [27 ± 2 per 100 μm^2^, *N* = 5; Welch’s *t*-test, using T distribution (DF = 2.5834) (two-tailed), the *P*-value = 0.24]. We also compared the thickness of the circular and longitudinal muscle cells between both cohorts of animals. Despite the differences in lengths of the colon, the average thickness of the circular smooth muscle was similar in both groups: control (128 ± 8 μm) and DTX-treated CAL-DTR colons (116 ± 6 μm) (*P* = 0.69; *N* = 5; Mann Whitney test). The longitudinal muscle was also not different between the two populations of mice: control (37 ± 2 μm) and DTX-treated CAL-DTR colons (32 ± 2 μm) (*P* = 0.42; *N* = 5; Mann Whitney test). Likewise, the diameter of the colons was not significantly different (proximal: 2.39 ± 0.22 vs. 2.35 ± 0.18 mm; mid: 2.13 ± 0.36 vs. 1.8 ± 0.18 mm; distal: 2.3 ± 0.25 vs. 2.02 ± 0.27 mm; *n* = 5 and 9, control and CAL-DTR, respectively, *p* = 0.116, two way ANOVA).

### Immunohistochemistry

**FIGURE 2:**
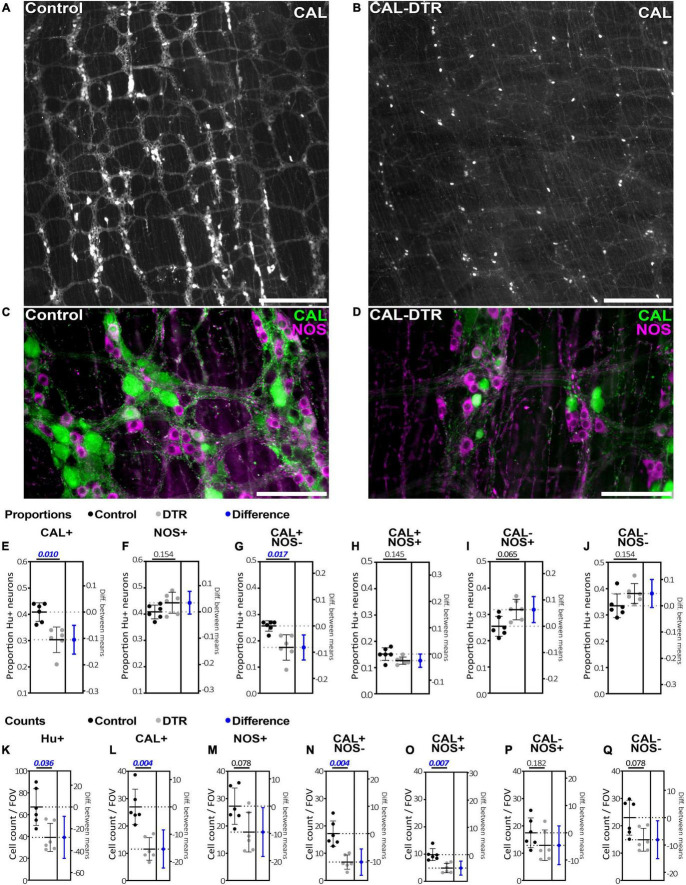
Calretinin and nitric oxide synthase immunolabeling in control and CAL-DTR colonic myenteric plexus. **(A,B)** Low power micrographs showing calretinin immunolabelling in control and CAL-DTR myenteric plexus. Calretinin immunolabelling appeared reduced in CAL-DTR compared to control, but CAL-IR nerve cell bodies can be seen persisting in CAL-DTR colon. **(C,D)** Higher power micrographs showing both calretinin and NOS immunolabelling in control and CAL-DTR colon. **(E–J)** Combinations of calretinin and NOS-IR myenteric nerve cell bodies, expressed as proportions of Hu-IR neurons. *P*-values for unpaired *t*-tests between control and CAL-DTR samples are shown above each graph. Blue markers indicate effect size, showing the mean and 95% confidence range of the difference between control and CAL-DTR groups. Significant differences have ranges that do not cross 0 difference (dotted line) on the right y axes. All statistical comparisons are independent sample *t*-tests, adjusted for multiple comparisons (Holm–Sidak method), and have *n* = 6 in each group. Control values are always noted before CAL-DTR values. The proportion of Hu-IR neurons that were CAL-IR **(E)**, but not NOS-IR **(F)** was significantly reduced in CAL-DTR mice, compared to control (calretinin: 41 ± 4% vs. 30 ± 5% *P* = 0.010; and NOS: 44 ± 4% vs. 41 ± 3%, *P* = 0.154). Among the four possible populations that can occur with calretinin and NOS double labelling, CAL-IR neurons lacking NOS **(G)** were significantly reduced as a proportion of Hu-IR myenteric neurons (26 ± 2% vs. 18 ± 5%; *P* = 0.017). **(H)** CAL-IR/NOS-IR neurons were not significantly reduced (15 ± 2% vs. 13 ± 1%; *P* = 0.145), nor were **(I)** NOS-IR neurons lacking calretinin (26 ± 4% vs. 32 ± 4%; *P* = 0.065), or neurons lacking both markers (**J**; 34 ± 5% vs. 38 ± 4%; *P* = 0.154). **(K–Q)** The average cell counts per field of view (FOV). All comparisons describe control values before CAL-DTR values, have *n* = 6 in each group and *P*-values adjusted for multiple comparisons using the Holm–Sidak method. **(K)** Hu-IR neurons were decreased by 42% in CAL-DTR colon compared to control (67 ± 20 vs. 39 ± 17 cells/FOV, *P* = 0.036). **(L)** Numbers of CAL-IR decreased significantly, by 57% (27 ± 9 vs. 12 ± 6 cells/FOV, *P* = 0.004), but not **(M)** NOS-IR neurons (27 ± 9 vs. 18 ± 9 cells/FOV, *P* = 0.078). Of the four neuronal populations arising from calretinin and NOS double labelling, CAL-IR neurons lacking NOS **(N)** showed the largest decrease in number in CAL-DTR colon, compared to control (61%; 17 ± 6 vs. 7 ± 3 cells/FOV, *P* = 0.004). **(O)** Neurons that were both CAL-IR and NOS-IR decreased 50% (10 ± 4 vs. 5 ± 2 cells/FOV, *P* = 0.007). **(P)** Neurons that were NOS-IR but lacked calretinin were not significantly changed in number (17 ± 7 vs. 13 ± 7 cells/FOV, *P* = 0.182). **(Q)** Nor were cells that lacked both calretinin and NOS (23 ± 9 vs. 15 ± 6 cells/FOV, *P* = 0.078). Calibrations, **(A,B)**, 500 μm; **(C,D)**, 200 μm.

Calretinin neuron ablation was assessed by quantifying Hu-IR, CAL-IR, and NOS-IR myenteric neurons in control and CAL-DTR animals (*n* = 6 each; Figure 2). At low power, calretinin immunoreactivity appeared generally less abundant in CAL-DTR mice compared to controls but immunoreactivity in nerve cell bodies clearly persisted (Figures 2A–D). To quantify this, an average 1352 ± 395 Hu-IR myenteric nerve cell bodies were sampled from each animal with equal weighting from the proximal, mid and distal thirds of the colon (control, total of 8,050 cells sampled, *n* = 6, CAL-DTR, total of 8,173 cells sampled, *n* = 6). In all following statistical comparisons of immunohistochemical data, *t*-tests are from independent samples, adjusted for multiple comparisons (Holm–Sidak), and control values are described before CAL-DTR values. CAL-DTR data presented are the normalised values corrected for by the change in colon length, see methods. On average, the number of Hu-IR cells contained within in 0.216 mm^2^ field of view (FOV) analysed was decreased by 42% in CAL-DTR colon compared to control (67 ± 20 vs. 39 ± 17 cells/FOV, *P* = 0.036; Figure 2K). In control colons, 41 ± 4% of Hu-IR myenteric neurons (*n* = 6) contained calretinin immunoreactive content (Sang and Young, 1996; Musser et al., 2015; Parathan et al., 2020). In CAL-DTR colons, 30 ± 5% of Hu-IR myenteric neurons contained calretinin (*P* = 0.010; Figure 2E). The numbers of CAL-IR neurons per FOV decreased significantly, by 57% (27 ± 9 vs. 12 ± 6 cells/FOV, *P* = 0.004; Figure 2L). NOS-IR neurons in CAL-DTR mice compared to controls were not significantly different as a proportion of Hu-IR neurons (44 ± 4% vs. 41 ± 3%, *P* = 0.154, Figure 2F), or in their numbers per FOV (27 ± 9 vs. 18 ± 9 cells/FOV, *P* = 0.078; Figure 2M). Analysis of calretinin neuron subtypes showed that the population of CAL-IR neurons that lacked NOS immunoreactivity was significantly decreased as a proportion of Hu-IR neurons (26 ± 2% vs. 18 ± 5%; *P* = 0.017; Figure 2G), and by 61% in their number per FOV (17 ± 6 vs. 7 ± 3 cells/FOV, *P* = 0.004; Figure 2N). CAL-IR neurons that contained NOS were not significantly reduced as a proportion of Hu-IR neurons (15 ± 2% vs. 13 ± 1%; *P* = 0.145; Figure 2H), while their numbers per FOV were significantly reduced by an average 50% (10 ± 4 vs. 5 ± 2 cells/FOV, *P* = 0.007). Neurons that lacked calretinin immunoreactivity but contained NOS-IR were not significantly different as a proportion of Hu-IR neurons (26 ± 4% vs. 32 ± 4%; *P* = 0.065; Figure 2I), nor in their numbers per FOV (17 ± 7 vs. 13 ± 7 cells/FOV, *P* = 0.182; Figure 2P). Neurons lacking both calretinin and NOS were also not significantly different both as a proportion of Hu-IR neurons (34 ± 5% vs. 38 ± 4%; *P* = 0.154; Figure 2J), and numbers per FOV (23 ± 9 vs. 15 ± 6 cells/FOV, *P* = 0.078; Figure 2Q). The number of myenteric ganglia around the circumference of each colonic region showed little difference between control and CAL-DTR preparations (Proximal: 38 ± 4 vs. 36 ± 4, Mid: 35 ± 2 vs. 32 ± 2, Distal: 39 ± 5 vs. 40 ± 4, *P* = 0.343 for main effect of genotype, 2-way ANOVA, *n* = 6). Taken together, the results suggest CAL-DTR colons had less myenteric neurons, explicable by a loss of calretinin neurons, with the largest effect seen in the population of calretinin neurons that lacked NOS. Detailed results of immunohistochemical analysis are listed in Table 1.

**TABLE 1:** Counts and proportions of Hu, CAL, and NOS immunoreactive myenteric nerve cell bodies in colon.

			Cell counts, uncorrected							Cell counts, corrected for gut length							Proportions					
		FOV samples	Hu±	CAL±	NOS±	CAL+/NOS−	CAL+/NOS+	CAL−/NOS+	CAL−/NOS−	Hu±	CAL±	NOS±	CAL+/NOS−	CAL+/NOS+	CAL−/NOS+	CAL−/NOS−	CAL±	NOS±	CAL+/NOS−	CAL+/NOS+	CAL−/NOS+	CAL−/NOS−
Prox	Control (*n* = 6)	47	73 ± 14	27 ± 5	29 ± 6	19 ± 5	8 ± 2	20 ± 5	27 ± 7	73 ± 14	27 ± 5	29 ± 6	19 ± 5	8 ± 2	20 ± 5	27 ± 7	0.36	0.39	0.25	0.11	0.28	0.36
	CAL-DTR (*n* = 6)	52	64 ± 23	17 ± 7	30 ± 15	10 ± 4	7 ± 3	23 ± 13	27 ± 10	48 ± 17	13 ± 5	23 ± 12	7 ± 3	5 ± 2	18 ± 10	21 ± 7	0.25	0.44	0.14	0.11	0.33	0.42
	P (multiple *T*-tests adjusted)		0.652	0.027	0.825	0.040	0.687	0.856	0.862	0.038	0.002	0.286	0.004	0.080	0.579	0.308	0.002	0.146	0.003	0.564	0.083	0.136
Mid	Control (*n* = 6)	34	72 ± 34	29 ± 17	29 ± 16	18 ± 11	11 ± 7	18 ± 10	26 ± 15	72 ± 34	29 ± 17	29 ± 16	18 ± 11	11 ± 7	18 ± 10	26 ± 15	0.42	0.42	0.26	0.16	0.26	0.32
	CAL-DTR (*n* = 6)	55	46 ± 23	12 ± 6	20 ± 10	7 ± 4	5 ± 2	15 ± 8	17 ± 8	35 ± 18	9 ± 5	15 ± 8	6 ± 3	4 ± 2	11 ± 6	13 ± 6	0.30	0.46	0.18	0.12	0.34	0.37
	P (multiple *T*-tests adjusted)		0.230	0.142	0.241	0.189	0.198	0.504	0.308	0.066	0.066	0.070	0.093	0.095	0.157	0.108	0.209	0.213	0.270	0.382	0.052	0.767
Distal	Control (*n* = 6)	39	56 ± 13	25 ± 4	24 ± 5	15 ± 3	10 ± 1	14 ± 5	16 ± 5	56 ± 13	25 ± 4	24 ± 5	15 ± 3	10 ± 1	14 ± 5	16 ± 5	0.46	0.44	0.28	0.18	0.26	0.28
	CAL-DTR (*n* = 6)	61	45 ± 21	17 ± 9	20 ± 10	10 ± 5	7 ± 4	13 ± 6	15 ± 6	34 ± 16	13 ± 7	15 ± 8	7 ± 4	5 ± 3	10 ± 5	11 ± 5	0.37	0.44	0.22	0.16	0.29	0.34
	P (multiple *T*-tests adjusted)		0.365	0.102	0.365	0.130	0.260	0.796	0.796	0.021	0.003	0.024	0.009	0.012	0.114	0.108	0.029	0.880	0.104	0.166	0.166	0.147
Whole	Control (*n* = 6)	120	67 ± 20	27 ± 9	27 ± 9	17 ± 6	10 ± 4	17 ± 7	23 ± 9	67 ± 20	27 ± 9	27 ± 9	17 ± 6	10 ± 4	17 ± 7	23 ± 9	0.41	0.41	0.26	0.15	0.26	0.34
	CAL-DTR (*n* = 6)	166	52 ± 22	15 ± 7	23 ± 12	9 ± 5	6 ± 3	17 ± 9	20 ± 8	39 ± 17	12 ± 6	18 ± 9	7 ± 3	5 ± 2	13 ± 7	15 ± 6	0.30	0.44	0.18	0.13	0.32	0.38
	P (multiple *T*-tests adjusted)		0.477	0.036	0.630	0.034	0.114	0.775	0.630	0.036	0.004	0.078	0.004	0.007	0.182	0.078	0.010	0.154	0.017	0.145	0.065	0.154

### Mechanical and Electrophysiological Recordings

**FIGURE 3:**
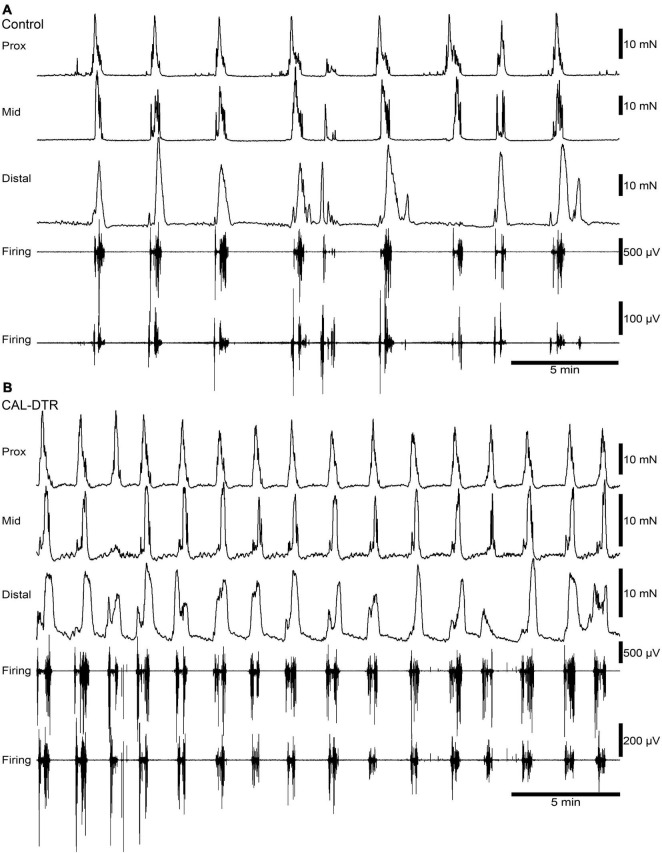
Ongoing colonic motor complexes and neurogenic spike bursts in control and CAL-DTR colon. **(A)** Typical high-amplitude propagating contractions in control colon, characteristic of CMCs. Neurogenic spike bursts are the myoelectrical correlate of the CMC, occurring with CMCs on a one-for-one basis. **(B)** Ongoing CMCs in CAL-DTR colon over the same time period shown in panel **(A)**. Note the increased CMC and neurogenic spike burst frequency.

**FIGURE 4:**
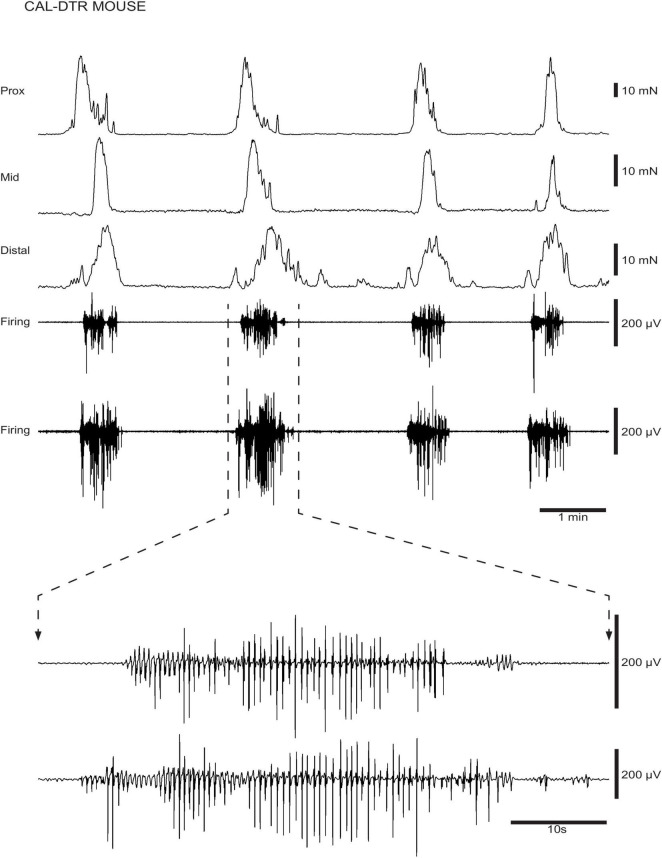
Ongoing colonic motor complexes (CMCs) and neurogenic spike bursts in CAL-DTR colon. CMCs and underlying neurogenic spike bursts are shown in a CAL-DTR mouse. The spike bursts are shown on expanded time scale showing the ∼2 Hz frequency of action potentials, on an expanded time scale.

**FIGURE 5:**
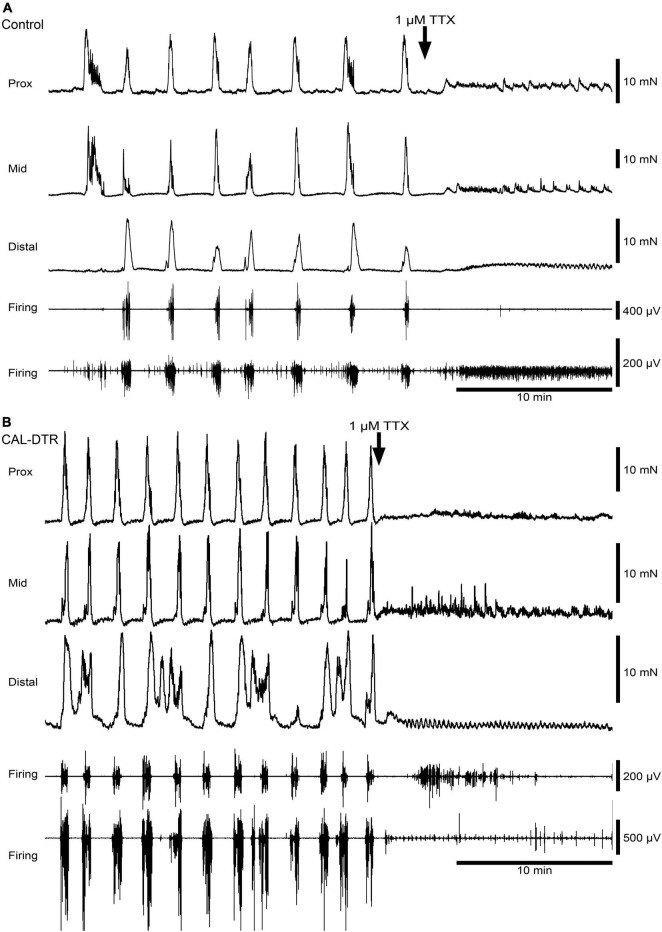
Blockade of colonic motor complexes (CMCs) and neurogenic spike bursts by tetrodotoxin in an isolated whole colon from control and CAL-DTR mice. **(A)** Tetrodotoxin (1 μM) promptly abolished ongoing CMCs and neurogenic spike bursts in both control preparations (4/4 animals) and **(B)** in CAL-DTR preparations (6/6 preparations), consistent with their neural origins. Rhythmic myogenic spiking and low-amplitude contractions were revealed several minutes after the abolition of neurogenic motor behaviour.

**FIGURE 6:**
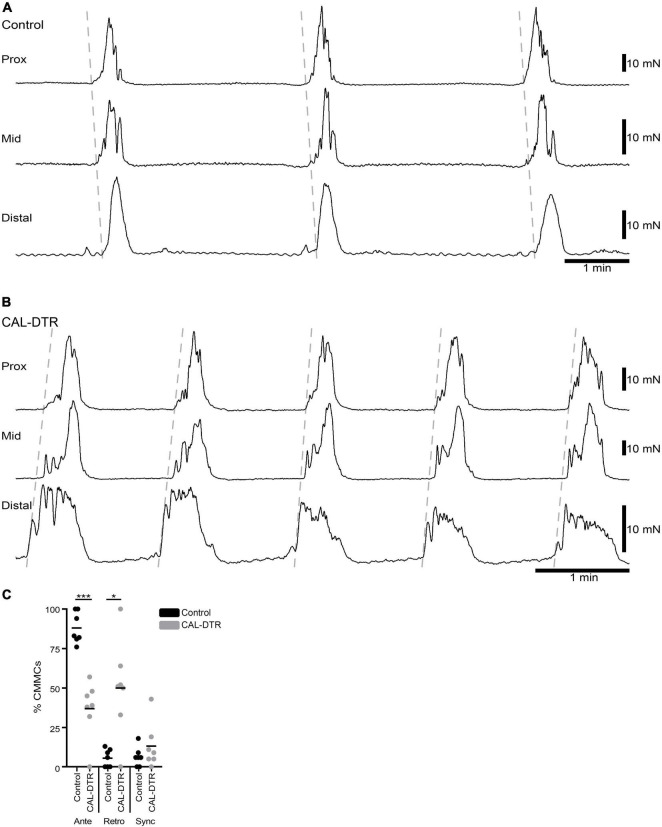
Propagation direction of colonic motor complexes (CMCs). **(A)** Example of CMCs in control colon showing a proximal to distal (anterograde) propagation. The vast majority of CMCs in control colon propagated in the anterograde direction (100/113 CMCs, *n* = 7). **(B)** Example of retrograde CMCs in CAL-DTR colon. Here, contractions were initiated in the distal colon and propagated towards the proximal colon. Approximately half of CMCs in CAL-DTR colons propagated retrogradely (75/169 contractions, *n* = 7). **(C)** Compared to control CMCs, those in CAL-DTR colon were significantly less likely to propagate anterogradely, and more likely to be retrograde contractions (2 way ANOVA Sidak post-test, ***P < 0.001, *P = 0.023, *n* = 7).

Mechanical and extracellular myoelectric recordings were performed on isolated tube preparations of whole colon, *in vitro* (control: *n* = 7; CAL-DTR: *n* = 7). All preparations had ongoing CMCs associated with “neurogenic spike bursts” (Hibberd et al., 2017; Figures 3, 4). CMCs and neurogenic spike bursts were immediately abolished by tetrodotoxin (10^–6^ M), indicating these activities were neurogenic (control: 4/4 preparations tested, CAL-DTR: 6/6 preparations tested; Figure 5). The vast majority of CMCs in control preparations propagated in the normal oral to aboral direction, appearing first in the proximal colon and migrating to the distal colon (100/113 CMCs, average 88 ± 10%, *n* = 7; Figure 6A). In contrast, CMCs in CAL-DTR colon lacked any preferential bias in direction of propagation direction: 75/169 CMCs were retrograde (average 50 ± 30%), arising first in distal colon and migrating to the proximal colon (Figure 6B); 69/169 CMCs migrated anterogradely (average 37 ± 18%, *n* = 7) and 25/169 CMCs had contractions that appeared synchronously, lacking discernible propagation direction (average 13.1 ± 14.5%). The proportion of antegrade CMCs were thus significantly decreased in CAL-DTR mice compared to controls (two way ANOVA, Sidak post-test, *P* < 0.001, *n* = 7) while retrograde contractions were increased (*P* = 0.023, *n* = 7). These data are summarised in Figure 6C.

**FIGURE 7:**
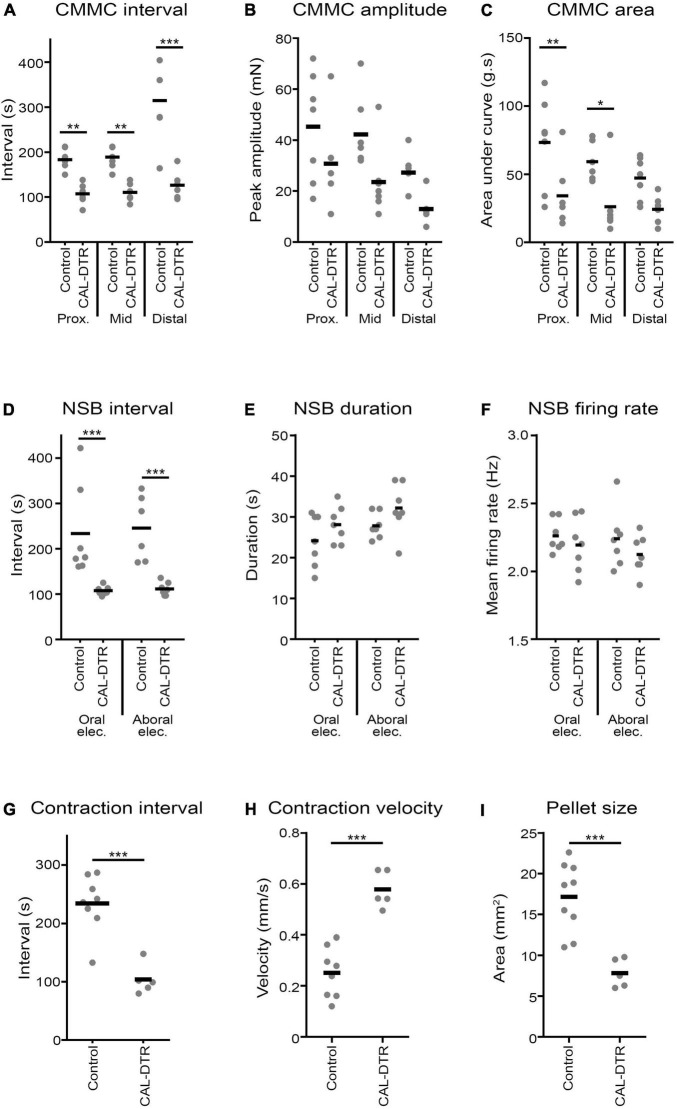
Properties of colonic motor complexes (CMCs), neurogenic spike bursts, and diameter-mapped preparations. **(A)** CMCs in CAL-DTR were significantly increased in frequency, showing smaller intervals between contractions in all regions of the colon. There was a significant main effect of genotype (*P* < 0.001, 2 way ANOVA, *N* = 7), in addition to multiple *post hoc* differences (Bonferroni) across different colonic subregions shown on the graph. Peak amplitudes **(B)** were significantly different across genotypes (genotype main effect, *P* = 0.001, 2 way ANOVA, *N* = 7), without significant *post hoc* differences in colonic subregions. **(C)** CMC contraction area under the curve was significantly different across genotypes (genotype main effect, *P* < 0.001, 2 way ANOVA, *N* = 7) and by *post hoc* comparison in the proximal and mid colon. **(D)** Similar to CMCs, neurogenic spike burst intervals were significantly reduced in CAL-DTR colon, compared to controls (genotype main effect, *P* < 0.001, 2 way ANOVA, *N* = 7). Neurogenic spike burst durations **(E)** were significantly different (genotype main effect, *P* = 0.047, 2 way ANOVA, *N* = 7), but not by *post hoc* test. The rate of action potential discharge within neurogenic spike bursts **(F)** was not significantly different by either genotype main effect or *post hoc* test. **(G)** As with CMCs and neurogenic spike bursts, propagating contractions in diameter-mapped preparations showed decreased intervals in CAL-DTR colon, compared to control. Contractions also propagated with higher velocity in CAL-DTR colon, compared to control **(H)**. **(I)** The maximum cross-sectional area of faecal pellets was substantially reduced in CAL-DTR colons, compared to control. ***P < 0.001, **P < 0.01, *P < 0.05, *N* = 7. NSB, neurogenic spike burst.

A conspicuous difference between control and CAL-DTR preparations was the observation that CMC frequency in CAL-DTR preparations was increased compared with controls. CMCs in control preparations had an average interval of 189 ± 24 s (mid-colon; *n* = 7) while CMCs in CAL-DTR colon occurred every 111 ± 19 s (*P* < 0.001, Bonferroni post-test, 2-way ANOVA; control: *n* = 7, CAL-DTR: *n* = 7; Table 2 and Figure 7A). The strength and duration of CMCs in CAL-DTR colon was reduced compared to control CMCs: peak amplitude, area under the curve and neurogenic spike burst durations were all significantly different (genotype main effects: *P* = 0.001, <0.001, <0.001, and 0.047, respectively, 2-way ANOVA). These data, including *post hoc* comparisons are detailed in Table 2 and Figure 7. Expectedly, neurogenic spike bursts, which underlie CMCs, were also more frequent in the CAL-DTR colon than in controls (Figure 7D).

**TABLE 2:** Characteristics of colonic motor complexes (CMCs) and neurogenic spike bursts.

	Mechanical activity												Myoelectric activity					
	CMC interval (s)			CMC amplitude (g)			CMC AUC (g.s)			CMC HPD (s)			NSB interval (s)		NSB duration (s)		NSB MFR (Hz)	
	Prox.	Mid	Distal	Prox.	Mid	Distal	Prox.	Mid	Distal	Prox.	Mid	Distal	Oral	Anal	Oral	Anal	Oral	Anal
Control (*n* = 7)	183 ± 23	189 ± 24	315 ± 81	4.5 ± 2.1	4.2 ± 1.4	2.7 ± 0.8	73 ± 33	59 ± 13	47 ± 16	18 ± 7	17 ± 5	17 ± 3	234 ± 102	246 ± 72	24 ± 6	28 ± 3	2.3 ± 0.1	2.2 ± 0.2
CAL-DTR (*n* = 7)	107 ± 21	111 ± 19	126 ± 31	3 ± 1.7	2.3 ± 1.4	1.3 ± 0.5	34 ± 23	26 ± 24	24 ± 10	12 ± 3	12 ± 2	21 ± 1	108 ± 10	112 ± 14	28 ± 5	32 ± 6	2.2 ± 0.2	2.1 ± 0.1
ANOVA	<0.001			0.001			<0.001			0.077			<0.001		0.047		0.283	
Post-test	0.003	0.002	<0.001	0.190	0.059	0.206	0.004	0.017	0.147	0.033	0.084	0.289	0.002	0.002	0.331	0.277	0.932	0.447

*Values represent mean ± s.d.; Abbreviations: NSB, neurogenic spike burst; AUC, area under the curve; HPD, half-peak duration; MFR, mean firing rate.*

### Natural Pellet Expulsion and Diameter Mapping

**FIGURE 8:**
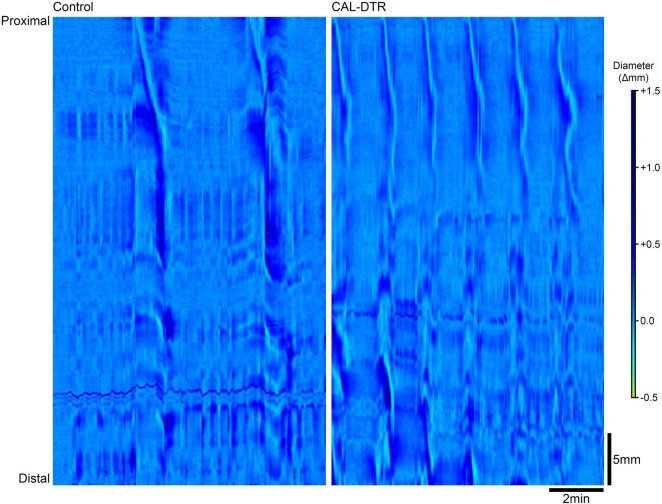
Propagating contractions in control and CAL-DTR colon. In these diameter maps, changes in gut diameter are shown with darker areas indicating increases in diameter while lighter areas indicate decreases in diameter. Both preparations contained pellets (control: seven pellets, CAL-DTR: four pellets). Time increases from left to right and the gut is orientated with proximal and distal ends from top to bottom. Control colon (left) shows 2 contractions in a 10 min period, compared to propagating contractions in the CAL-DTR colon (right). Note the higher frequency in CAL-DTR colon, with 6 contractions in the same 10 min period. All contractions arise from the most proximal end of the gut.

Isolated whole colon preparations containing natural pellets were setup and video recorded for spatiotemporal mapping, *in vitro* (control: *n* = 9, CAL-DTR: *n* = 5). Spontaneous expulsion of endogenous pellets were also counted. Control and CAL-DTR preparations were initially found to contain 6 ± 2 and 4 ± 2 pellets on average, respectively (*P* = 0.148, independent samples *t*-test, control: *n* = 9, CAL-DTR: *n* = 5). Pellets in CAL-DTR preparations were significantly smaller than those in control preparations (maximum cross-sectional area 7.8 ± 1.8 vs. 17.2 ± 4.2 mm^2^, respectively, *P* = 0.005, independent samples *t*-test, CAL-DTR: *n* = 5, control *n* = 9; Table 3). There was no significant difference in the proportion of pellets expelled between preparations (44 ± 32% vs. 55 ± 55% in control and CAL-DTR, respectively, *P* = 0.508, independent samples *t*-test, control: *n* = 9, CAL-DTR: *n* = 5). However, propagating contractions in CAL-DTR preparations occurred more frequently than in control preparations (control interval: 234 ± 49 vs. CAL-DTR: 104 ± 26 s, respectively, *P* < 0.001, independent samples *t*-test, control: *n* = 9, CAL-DTR: *n* = 5). Representative diameter maps showing different contraction intervals are shown in Figure 8. Propagating contractions in CAL-DTR preparations also had a higher velocity compared to control (control: 0.25 ± 0.10 vs. CAL-DTR: 0.58 ± 0.08 mm.s^–1^, *P* < 0.001, independent samples *t*-test, control: *n* = 9, CAL-DTR: *n* = 5; Table 2). Unlike recordings of CMCs using force transducers, the direction of propagation along colons that contained pellets from control and CAL-DTR mice were similar (Figure 8). All propagating contractions in CAL-DTR preparations arose from the proximal colon and travelled anterogradely (control: 65/66 contractions CAL-DTR: 59/59 contractions, *n* = 9 and 5, respectively). These data are summarised in Table 3.

**TABLE 3:** Characteristics of endogenous pellets and propagating contractions during recordings for spatiotemporal mapping.

	Pellet characteristics			Propagating contraction characteristics		
	Diameter (mm)	Length (mm)	Area (mm^2^)	Interval (s)	Velocity (mm.s^–1^)	Pellet expulsion (%)
Control (*n* = 9)	3.3 ± 0.4	6.8 ± 1.1	17.2 ± 4.2	234 ± 49	0.25 ± 0.10	55 ± 55
CAL-DTR (*n* = 5)	2.3 ± 0.3	4.2 ± 0.6	7.8 ± 1.8	104 ± 26	0.58 ± 0.08	44 ± 32
*T*-test	<0.001	<0.001	<0.001	<0.001	<0.001	0.508

## Discussion

In this study, it was found that when diphtheria toxin was administered to mice that expressing human diphtheria toxin receptors in calretinin neurons, there was a reduction in the total population of colonic myenteric calretinin neurons by about 25%, with the most significant losses among calretinin neurons that lacked co-expression of NOS. Unlike general neuronal loss incurred by chemotherapeutics, inflammation and hereditary hypoganglionosis which all decrease CMC frequency *in vitro*, a specific reduction of calretinin neurons in the present study led to increased CMC frequencies. Other characteristics of neurogenic motility were modified, including increased propagating contraction frequency, the directionality of CMC propagation, velocity of propagation and contraction forces. Thus, enteric neurons that express calretinin may play a role in controlling characteristics of CMCs which underlies propulsive motility and transit in mouse colon (Spencer et al., 2018).

### Calretinin Neuron Ablation

Chemogenetic ablation using targeted diphtheria receptor expression has been used to study immune signalling in the gut (Dunkin et al., 2017; Kang et al., 2019) and inducible diphtheria toxin subunit A has been used to ablate enteric glia (Rao et al., 2017). The latter showing a slightly higher ablation efficacy of 66 and 74% losses of S100β-IR myenteric and intramuscular glial cells in the small and large intestine, respectively. The targeted DTR method to ablate neurons in the ENS has not previously been used and represents an exciting new step forward to probe the functional roles of enteric neurons. The statistically significant results of the immunohistochemical analysis in the present study were consistent with most expected effects of calretinin ablation. That is, there was a significant reduction in numbers of Hu-IR neurons, indicating losses of myenteric neurons and not just calretinin protein expression. There were also loss of calretinin neurons both as their proportion of Hu-IR neurons and in their average numbers per FOV along the colon (see Figure 2 and Table 1). Among calretinin neurons the largest effect (61%) was seen among those that lacking NOS, and they showed reductions both as a proportion of Hu-IR neurons and in their numbers per FOV. Calretinin neurons that contained NOS were reduced in their counts (50%) but not as a proportion of Hu-IR neurons, possibly in part due to the larger effect seen in those lacking NOS. Whether the reduced effect was related to NOS expression is unclear (Sandgren et al., 2003; Lin et al., 2004).

The effect on Hu-IR neuron counts (losses of 28 cells per FOV), represents a 42% reduction, which is similar to the total proportion of CAL-IR neurons in control preparations of ∼41%. However, ablation of CAL-IR neurons was not complete: the average effect size among the CAL-IR populations (average losses of 15 cells per FOV) represented just over half of the reduction in Hu-IR neurons (28 cells per FOV). Notwithstanding neurochemical plasticity (*de novo* or increased calretinin expression in surviving myenteric neurons), the discrepancy in these effect sizes may represent evidence of an effect of DTX administration on other neurons, either by off-target *cre*-driven expression of the diphtheria receptor or secondary to a dependence on ablated calretinin neurons. The likelihood of the former possibility cannot be completely ruled out but is allayed by previous characterisation of the *cre*-driven expression of eYFP in the same Calb2-IRES-Cre mice used in the present study to generate CAL-DTR mice. In these mice, 97% of eYFP expression occurred in calretinin-immunoreactive neurons (Hibberd et al., 2018a). However, a subset of calretinin neurons (likely the morphologically Dogiel type II population that also contain CGRP; Furness et al., 2004) represent the exclusive source of nerve growth factor among mouse colonic myenteric neurons (Petrie et al., 2015). Thus, calretinin neuron ablation may have led to a loss of NGF signalling with secondary effects on other neurons. However, survival of trkA-expressing enteric neurons was not solely dependent on NGF signalling (Petrie et al., 2015), suggesting involvement of other factors (Liu, 2018). Nevertheless, the possibility of off-target neuronal losses cannot be ruled out despite the apparent selectivity of the statistically significant effects for calretinin-expressing neurons, and this should be considered in interpreting results of the present study.

### Colonic Motor Complex Contraction Strength

Decreased contraction strength is an expected consequence of calretinin neuron ablation. Smooth muscle contraction of the murine colon during the CMC is predominantly driven by acetylcholine and tachykinins (Bywater et al., 1989; Bush et al., 2000; Brierley et al., 2001). Circular muscle motor neuron nerve terminals that express choline acetyltransferase, vesicular acetylcholine transporter, and substance P also express calretinin the mouse colon (Sang and Young, 1996; Sang et al., 1997; Sang and Young, 1998). Thus, decreased strength of contractions observed in CAL-DTR mice may be explicable by loss of excitatory input to the smooth muscle.

### Direction of Contraction Propagation

Colonic motor complexes without intraluminal content propulsion are preferentially anterograde but can occur retrogradely, or synchronously along the gut (Powell and Bywater, 2001; Powell et al., 2002; Powell et al., 2003; Keating and Spencer, 2010; Copel et al., 2013; Costa et al., 2020). CMCs in the CAL-DTR colon were characterised by a loss of anterograde preference and a preponderance of CMCs that propagated in the retrograde direction. This suggests calretinin enteric neurons may play a role in regulating CMC polarity.

Whilst CMCs do not require intraluminal content, movement of intraluminal content may involve an additional mechanism whereby local polarised neural circuits are sequentially activated by content in a neuromechanical cycle (Dinning P. G. et al., 2014; Costa et al., 2015). Indeed, the polarity of intraluminal fluid (Roberts et al., 2007; Roberts et al., 2008; Costa et al., 2013; Sasselli et al., 2013; Wafai et al., 2013; Welch et al., 2014; Barnes and Spencer, 2015; Balasuriya et al., 2016; McQuade et al., 2016; Robinson et al., 2017; Vincent et al., 2018; Israelyan et al., 2019; Leembruggen et al., 2020), and pellet propagation, is virtually always anterograde (Barnes et al., 2014; Balasuriya et al., 2016; Diss et al., 2016; McQuade et al., 2017; Hibberd et al., 2018a; McQuade et al., 2018). In contrast to the loss of anterograde preference in non-propulsive CMCs in CAL-DTR colon in the present study (Figure 6), there was no difference in propagating contraction direction between control and CAL-DTR colon during natural pellet expulsion: nearly all contractions in both groups propagated anterogradely. Thus, the polarity of colonic propulsive motility withstands the losses of calretinin neurons imposed in the present study. Short distances of pellet “retropulsion” have been observed (Brann and Wood, 1976; Heredia et al., 2013), however, we are unaware of any reports of reversal of colonic propulsion *in vitro* or *in vivo*, such that pellets located in the distal colon may be propelled and eventually expelled from the proximal colon. We speculate that calretinin neurons contribute more heavily to non-propulsive CMC polarity, than to the robust polarity of propulsive contractions, possibly due to additional recruitment of local polarised circuits. Indeed, normal anterograde propulsion in the gut is fundamental to life and interruption of anterograde propulsion, as occurs with deletion of choline acetyl transferase from the ENS (Johnson et al., 2018) or in aganglionosis (Pichel et al., 1996; Shen et al., 2002), may be lethal.

### Colonic Motor Complex Frequency, Loss of Enteric Neurons, and Intrinsic Primary Afferent Neurons

Understanding which neurons in the ENS are responsible for the frequency of neurogenic contractions along the colon and their direction of propagation has been a major unresolved issue. A major observation of this study was that ablation of calretinin neurons increased CMC frequency. This is opposite to the effects observed in murine experimental models that cause non-specific (cholinergic and non-cholinergic) myenteric neuronal losses in the colon. These include chemotherapeutics (Wafai et al., 2013; McQuade et al., 2016; McQuade et al., 2017; McQuade et al., 2018; Stojanovska et al., 2018; McQuade et al., 2019), colitis (Robinson et al., 2017; Hofma et al., 2018), and disrupted endothelin-3 signalling (Ro et al., 2006; Roberts et al., 2008; Barnes and Spencer, 2015), all of which decrease CMC frequency. Pharmacological blockade of nitric oxide signalling is well known to increase the CMC frequency (Fida et al., 1997; Powell and Bywater, 2001), while the effect of genetic knockout is equivocal (Dickson et al., 2010; Spencer, 2013). Thus, loss of nitric oxide signalling provides a possible explanation for the increases in CMC frequency in the present study. However, this seems unlikely, since although their numbers were halved (Figure 2O), the loss of NOS-IR calretinin neurons as a proportion of all Hu-IR neurons was small and not statistically significant (15 ± 2% vs. 13 ± 1%; *P* = 0.145; Figure 2H).

The most significantly reduced cell population in CAL-DTR mice were CAL-IR neurons lacking NOS, which is consistent with the neurochemistry of Dogiel type II IPANs (Furness et al., 2004). About 99% of myenteric Dogiel type II neurons (IPANs) express calretinin (Furness et al., 2004), and few express NOS (Hibberd et al., 2018b). IPANs have been suggested to initiate CMCs (Bayguinov et al., 2010; Smith and Koh, 2017), possibly *via* mucosal 5HT release (Heredia et al., 2013; Smith and Gershon, 2015). Regardless of whether IPANs *initiate* CMCs, there is direct evidence that IPANs participate in the neuronal firing pattern underlying CMCs (Spencer et al., 2018). There are few studies in which experimental interventions causing neuronal loss or loss of function result in an increase in CMC frequencies. Those that have include P2 × 2 receptor subunit knockout (DeVries et al., 2010) and NaV1.9 knockout (Copel et al., 2013). Interestingly, virtually all Dogiel type II neurons in mouse colon express P2 × 2 receptors, and a subset express NaV1.9 (Copel et al., 2013). Conversely, specific enhancement of IPAN excitability by luminal administration of a neuroactive microbiota has led to decreased intestinal motor complex and colonic propagating contraction frequencies (Wu et al., 2013; West et al., 2017). Thus, the effects on CMC frequency observed with calretinin neuron ablation in the present study may reflect disruption of colonic IPAN function and/or the cells that receive their synaptic outputs. We recently characterised myenteric varicosities arising from IPAN “baskets”–dense clusters of varicosities that represent putative synaptic outputs of IPANs in mouse colon (Smolilo et al., 2019). Most IPAN varicosities (69%) contained calretinin without NOS. Additionally, 35% of IPAN baskets surrounded CAL-IR myenteric nerve cell bodies (Smolilo et al., 2020). Confirmation or rejection of a role in CMC pacing by IPANs awaits more specific pharmacological or neurogenetic targeting of this population.

In a previous study, channelrhodopsin-mediated activation of calretinin neurons evoked premature CMCs and colonic propulsion (Hibberd et al., 2018a). It is interesting then that a reduction of the calretinin neuron population may lead to increases in the frequency of CMCs. We speculate that activation of calretinin neurons is sufficient but not necessary to evoke CMCs. That is, neurons that lack calretinin may also trigger CMCs. In support of this, CALR-IR myenteric neurons represented only 28% of directly mechanosensitive neurons in the mouse distal colon, despite the majority of mechanosensitive neurons being cholinergic (Mazzuoli and Schemann, 2012). This implies numerous other cholinergic neurons capable of directly transducing mechanical stimuli—a major stimulus of CMCs, and is consistent with redundancy of functions in the ENS, which is capable of withstanding major neuronal deficits whilst still showing neurogenic motility patterns (Ro et al., 2006).

### Colonic Dimensions and Inflammation

Calretinin neuron losses coincided with a significantly reduced colonic length in the present study. Inflammatory states typically show decreased colonic length (Campaniello et al., 2017; DiCello et al., 2018; Hofma et al., 2018), but indications of inflammation were not present in CAL-DTR colons. Interestingly, a lack of inflammation was also seen using an inducible diphtheria toxin subunit A (DTA) neurogenetic approach to ablate enteric glial cells (Rao et al., 2017), despite inflammation arising in other approaches to ablate glia (Bush et al., 1998). In comparison, they concluded the lack of inflammation following ablation of glial cells in their study was attributable to the DTA method. This is compatible with the finding in the present study that neuronal cell ablation can be achieved using the related DTR approach, without inducing inflammation. Other mouse models that have shown decreased colon length in the absence of overt inflammation include disruption of toll-like receptor 4 signalling (Riehl et al., 2015), and knockout of adenosine A3 receptors (Ren et al., 2011). Whether these mechanisms relate changed colonic length and calretinin neuron losses in the present study remains to be shown.

## Conclusion

The effects of calretinin ablation in the present study suggest roles in regulating the frequency, force and direction of migration of CMC contractions along the mouse colon. The present study implicates enteric calretinin neurons in the overall regulation of colonic motility and gross morphological structure.

## Data Availability Statement

The original contributions presented in the study are included in the article/supplementary material, further inquiries can be directed to the corresponding authors.

## Ethics Statement

The animal study was reviewed and approved by Animal Welfare Committee of Flinders University and Animal Studies 81 Committee at Washington University School of Medicine.

## Author Contributions

JF, TH, JL, PY, and LT performed the experiments and analysed the data. HH, NS, JF, and TH developed the study design and wrote the manuscript. All authors read and approved the final version of the manuscript.

## Conflict of Interest

The authors declare that the research was conducted in the absence of any commercial or financial relationships that could be construed as a potential conflict of interest.

## Publisher’s Note

All claims expressed in this article are solely those of the authors and do not necessarily represent those of their affiliated organizations, or those of the publisher, the editors and the reviewers. Any product that may be evaluated in this article, or claim that may be made by its manufacturer, is not guaranteed or endorsed by the publisher.
